# Is Research in Simulation as Accessible as Traditional Clinical Research? A Review of the ‘Association for Simulated Practice in Healthcare’ Conference

**DOI:** 10.7759/cureus.9798

**Published:** 2020-08-17

**Authors:** Ben Gabbott, Philip Beak, Michael Stoddart, Rebecca V Morgan, Dean Malik, Deborah M Eastwood

**Affiliations:** 1 Trauma and Orthopaedics, Royal National Orthopaedic Hospital Stanmore, London, GBR; 2 Trauma and Orthopaedics, Kingston Hospital, London, GBR; 3 Paediatric Orthopaedics, Royal National Orthopaedic Hospital Stanmore, London, GBR

**Keywords:** simulation medicine, skills and simulation training, higher education medical training, research methodology

## Abstract

Background

Meta-analysis of simulation teaching has shown to be an effective teaching methodology. The Association for Simulated Practice in Healthcare (ASPIH) annual international, multidisciplinary conference is recognised as the leading UK meeting for simulation-based education. We hypothesise that simulation-based research presented at this conference is currently less accessible than more traditional clinical research presentations.

Method

We reviewed the abstracts of all research presented at the 5^th^ ASPIH Conference, 2014 and then utilised the Bhandari methodology to assess whether an abstract had subsequently been published in a peer review journal. Our secondary aim was to assess for recurring themes that may predict publication.

Results

Twenty-seven of 197 (14%) abstracts presented at the 2014 meeting were subsequently published. The mean lead time to publication from the conference was 23 (2 - 61) months. Two positive predictive factors for publication were oral presentations (vs poster), and a Kirkpatrick level above 1.

Conclusion

The publication rate for abstracts from respected clinical conferences is 30%, but the publication rate for ASPIH abstracts is significantly below this. The potential reasons for this may include a lack of simulation specific journals. Authors should aim to publish simulation-based research in peer reviewed publications to help progress the role and the value of simulation in medical education.

## Introduction

Simulation training is the creation of a ‘true to life’ learning environment that mirrors real life work scenarios, allowing us to learn from our experience. It includes the testing of a multitude of competencies including decision making attributes and practical skills. Simulation has been widely accepted across medical education programmes in the UK, with national guidelines now promoting its use with compulsory training sessions in a simulated environment for most healthcare workers [1]. Meta-analysis of simulation teaching has shown it to be an effective teaching methodology [2]. It is shown to be associated with large positive effects when compared to no intervention, and small to moderate effects when compared to traditional didactic type teaching. It has also been shown to improve learner competence and patient safety [2,3]. However, as a specialty branch of medical education, it is still in its relative infancy.

International conferences provide opportunities for like-minded professionals to exchange ideas and data. It is widely accepted that the gold standard for research studies is that the results are submitted to peer review and published as a journal article [4]. Published abstracts of conference podium presentations often contain less detail and the review process is less stringent than for paper publication. Despite this, an oral presentation is still highly regarded. Presentations allow the dissemination of new initiatives and early results more quickly than written publication, stimulating debate, and guiding others’ research. Previous studies have suggested that up to 63% of specialty textbook chapters have included results from oral presentation [5].

More recently studies have used the conversion rate of conference research presentations to journal publications, to assess the quality of a conference. The mean percentage of oral presentations that go onto be published in peer review journals from a respected conference with a clinical focus is approximately 30% [6,7].

The Association for Simulated Practice in Healthcare (ASPIH) annual international, multidisciplinary conference is recognised as the leading UK meeting for simulation-based education. It is attended by approximately 500 delegates and is accredited by the Royal College of Anaesthetists. Attendees respond positively with respect to the benefits on their own simulation teaching [8]. However, anecdotally, some delegates have reported issues accessing the data from simulation conferences which could potentially have a detrimental effect on both the set up and development of local simulation centres and also on the direction of future research. Our hypothesis is that simulation-based research is currently less accessible than more traditional clinical research. We define accessibility as referenceable, published papers, in peer reviewed journals.

Aim

Our primary aim was to calculate the percentage of research presented at the ASPIH conference which was subsequently published in a peer reviewed journal. Our secondary aim was to analyse all the abstracts from the ASPIH conference to identify any factors that predicted progress to publication.

## Materials and methods

We reviewed the abstracts of all research presented on the podium or as a poster at the 5th ASPIH Conference, 2014, Nottingham. The 2014 conference was selected as it gave a five year follow up period for research to be published. This is in keeping with similar, previous studies [5,6].

A database was created by four members of the research team (BG, DM, PB, RM). Extrapolated data included lead authors, type of presentation, type of research, theme, significance of results (as reported by the author), number of sites, number of participants, and publication details. Full data is available on request.

After data extrapolation, the papers were scored using the Kirkpatrick Level [9], a previously validated scoring system to assess the effectiveness of an educational intervention (Table 1).

**Table 1:** Kirkpatrick’s impacts on learning outcomes framework

Key Outcomes - Kirkpatrick Evaluation Levels	
Outcomes	Descriptor
1. Reaction	Participant’s views on the learning experience, its organization, presentation.
2. Learning of Knowledge or skills	For knowledge, this relates to acquisition of concepts, procedures and principles; for skills, this relates to the acquisition of thinking/problem solving, psychomotor, and social skills.
3. Learning / Behavioral Change	The transfer of learning to the workplace (i.e. surgical practice) or willingness of learners to apply new knowledge and skills.
4. Change in the system/organizational practice or Benefits to patients / communities	Wider changes in the organization, attributable to the practice of MTT OR benefits to patients / wider public/ communities as a result of faculty development.

To assess whether an abstract had been published following presentation, the Bhandari methodology was utilised as described in previous similar projects [5]. This involves the use of a search engine to identify related research using key word and author names. The search took place in December 2019 using Pubmed as the search engine, in keeping with similar previous research [6,7]. Once the papers were identified, the authors then extracted relevant information about the research itself and its subsequent publication details.

To look for recurring themes that may predict publication, analysis was undertaken with IBM SPSS Statistics version 25 (IBM Corp., Armonk, NY). Descriptive statistics were used for categorical data. The study assumed that p values of less than 0.05 were significant.

## Results

In total 197 abstracts were reviewed; 41% were oral presentations (Table 2). ‘Other types of research’ included short communications and industry/technology presentations looking at specific pieces of simulation equipment. For analysis, these were included as ‘Oral Presentations’ as they were given in a similar method, on a podium with a presentation, bringing the total to 57%.

**Table 2:** Demographics of the abstracts analysed and the publication rate

Demographics	
Total Number of Abstracts	197
No of Oral Presentations	81 (41%)
No of Poster Presentations	84 (43%)
No of ‘Other Types’ of Abstract	32 (16%)
No of Abstracts Published	27 (14%)

Of the 197 abstracts, 27 (14%) were subsequently published in PubMed listed journals (Table 3). The mean lead time to publication from the conference was 23 (Range 2 - 61) months. Five of 27 (18.5%) abstracts had been published prior to the ASPIH Conference.

**Table 3:** Journals in which ASPIH research was published ASPIH: Association for Simulated Practice in Healthcare

Journal	No of Publications	Open Access	Free Publication
Acta Orthopaedica	4	Y	N
International Journal of Surgery	3	Y	N
BMC Medical Education	2	Y	N
PLOS ONE	2	Y	N
Nurse Education Today	1	Y	N
British Journal of Hospital Medicine	1	Y	N
Clinical medicine	1	Y	Y
Anaesthesia	1	Y	N
BMJ simulation & technology enhanced learning	1	Y	N
Contemporary Ergonomics and Human Factors	1	Y	N
Resuscitation	1	Y	N
Journal of Surgical Education	1	Y	Y
World Journal of Surgery	1	N	Y
Annals Royal College Surgeons England	1	Y	N
British Journal of Anaesthesia	1	Y (delayed)	Y
Diagnostic and Prognostic Research	1	Y	N
Journal of Inter-professional Care	1	Y	N
Dementia (London)	1	Y	N

Factors that were tested to see if they were predictive of subsequent peer reviewed publication are detailed in Table 4. Two positive predictive factors for publication were identified to be significant. Firstly, abstracts that were podium presentations were significantly more likely to go on to be published (p=0.002). Secondly, a Kirkpatrick score greater than level 1 was significantly more likely to result in publication (p=0.017).

**Table 4:** Factors that may predict publication *Group 1 compared to Group 2,3,4

Predictive Publication Factors	P-Value
Oral vs Non-Oral Presentation	0.002
Kirkpatrick Level*	0.017
Significant results vs not significant results	0.067
Single vs Multi Centre Research	0.171

If the presented abstracts contained statistically significant results, the positive prediction for publication approached significance (p=0.067). Seniority of the presenting author had no effect on the prediction of publication; students showed a non-significant trend when compared to non-students (p=0.33). A multicentre trial design had no significance to the publication rate when compared to single site studies.

Of the studies that did progress to publication, 59% (10) demonstrated significant variance from the original published abstract. These changes included more authors, a change in number of participants, longer follow up, and being included as part of larger review with further interventions and analysis.

## Discussion

Over the last 20 years, the field of medical education has grown rapidly and significant developments have taken place. Trainees are now faced with an ever-increasing number of technical competencies to ‘sign off’, with rapidly reducing opportunities to practice and learn these skills in real life. This is further complicated by an increasing focus being placed on patient safety and risk awareness. Unfortunately, Halsteds principle of ‘see one, do one, teach one’ does not attain modern day safety or teaching standards, and many have called for the replacement of the ‘apprenticeship’ learning model with a more modern format [10].

Simulation offers a potential remedy in that students can be placed in realistic, stressful, and complex environments. Their skills and knowledge can be tested to failure, with the knowledge that the possibility of patient harm has been removed. Furthermore, simulation promotes feedback, reflection and discussion, encompassing the modern idea of experiential learning. It is a continuous, active process engaging everyone involved whether active or observing. As such, there is now an abundance of evidence supporting its use in education. Its positive results on skill acquisition, education, and non-technical skills are also widely accepted [11,12].

However, as with all developing methodologies, there are limitations. There is still a great degree of variance from one simulation centre to the next, with differing set ups, equipment, and candidates at each session. Simulation, best methods of implementation, and its role in the curriculum are also still debated [13,14]. Over recent years, there have also been great advances in the use of technology to promote realistic environments but with this comes an increasing financial cost. Simulation training is not cheap and the purchase of state of the art simulation equipment is often based on little evidence of its educational benefit.

Our research suggests that the publication rate of abstracts presented at the ASPIH conference is lower than the publication rates of other conferences that focus on clinical research. This may have a detrimental effect on the progression of simulation teaching, as the data presented at the conference may not be easily accessible by other educational establishments. Oral presentations should be seen as a stepping-stone to peer reviewed publications. If a research project stops once it has been presented, the only data to reference is the published abstract. A 250-word abstract submitted months before presentation, often lacks sufficient detail to allow the research to be replicated or validated by other professionals. Abstracts are also often submitted prior to completion of the study, meaning that the data they contain frequently varies from the data that is presented in the final oral presentation and the subsequent publication. This can be seen in our results, with 58% of final publications containing data and study details that vary significantly from the original published abstract.

The reason behind the poor publication rate is likely to be multifactorial, and further research is required to investigate this further. There are generally more difficulties encountered with researching educational methodologies when compared to more traditional research. These include the difficulties in quantifying learning and behavioural changes, results are often qualitative and opinion based.

There are also significantly fewer journals specific to simulation and education, giving less opportunity to be published. This is highlighted by re-comparing our results back to the two previously quoted benchmark studies [6 + 7]. Al-Hourani et al. studied an orthopaedic conference and found that nine out of the top 10 journals which published work presented at the conference were specific orthopaedic journals, in comparison to the ASPIH conference, which published work across 18 different journals, however, zero of these were dedicated to simulation [7].

The lack of specific simulation-based journals does pose a potential challenge, to our knowledge we know of four journals solely focussed on simulation. Ideally, simulation focussed journals will become as established as their clinical research counterparts, as demand for them increases. However, it takes time to establish new journals or publication portals and meanwhile, the demand needs to be created by those working in the specialty. With this in mind authors should continue to submit research to the few simulation journals that are currently available, or to those journals that have a secondary interest in such research.

We acknowledge that by comparing a developing educational specialty to established clinical specialties is potentially unfair. Clinical medicine has been researched and published for more than a hundred years, by thousands of professionals across the world. Simulation has been widely accepted across medical training programmes in the UK and is considered, by many, to be the keystone of post graduate teaching. It is therefore of paramount importance that we try to attain the same standards that we would expect to see in our clinical work to this ‘new’ specialty also. Lots of those working in simulation have trained as scientists or clinicians, or at least previous experience of research, so they etunderstand the importance of research standards and subsequent publication. Furthermore, simulation education is not a standalone specialty; it will always be inherently linked to the clinical specialty that is being taught. The end result of a clinicians simulation education, unfortunately, will be scrutinised in a much more traditional way like that of traditional clinical research.

With respect to our secondary aim, two factors were identified which increased the likelihood of publication. These were an ‘Oral Presentation’, as opposed to a poster presentation, and a presentation scoring a Kirkpatrick level above 1. An oral presentation is considered to supersede a poster, and equally the higher the Kirkpatrick level the more effective the education intervention. It is therefore unsurprising that the two factors which indicated better quality research were more likely to progress to publication. 

Our research has its limitations, only one search engine was used to check for publication and although this was in keeping with similar research in the area, there is still potential for research to be published outside of PubMed. To investigate this further, other conferences should also be investigated for their publication rates to confirm or refute the suggestion that simulation research is less accessible.

## Conclusions

The ASPIH conference and other similar conferences offer a platform to promote research and drive expertise forward. However, research projects should not stop after the ‘Closing Comments’ lecture of a conference. With increasing responsibility being placed on simulation teaching, combined with increasing costs of simulation suites, it is our responsibility as researchers in the medical education community, to ensure our efforts are appropriately supported with medical evidence. This starts with the publication of our own research in peer reviewed journals. With regards to the research itself, all authors should aim to conduct research with longitudinal follow up, in keeping with high Kirkpatrick levels. All methodologies should aim to be published with enough evidence to allow other centres to replicate the results. With this, we can aim to improve and drive simulation teaching forwards uniformly across the country. 
